# Potential of emodepside for vector-borne disease control

**DOI:** 10.1186/s12936-025-05250-8

**Published:** 2025-01-11

**Authors:** Pattarapon Khemrattrakool, Thitipong Hongsuwong, Theerawit Phanphoowong, Patchara Sriwichai, Kittiyod Poovorawan, Joel Tarning, Kevin C. Kobylinski

**Affiliations:** 1 Mahidol Oxford Tropical Medicine Research Unit, Faculty of Tropical Medicine, Mahidol University, Bangkok, Thailand; 2 Department of Medical Entomology, Faculty of Tropical Medicine, Mahidol University, Bangkok, Thailand; 3 Department of Clinical Tropical Medicine, Faculty of Tropical Medicine, Mahidol University, Bangkok, Thailand; 4 Centre for Tropical Medicine and Global Health, Nuffield Department of Clinical Medicine, University of Oxford, Oxford, UK

**Keywords:** Emodepside, Ivermectin, *Anopheles dirus*, *Aedes aegypti*, Mosquito, Survival

## Abstract

**Background:**

Emodepside is an anthelmintic used in veterinary medicine that is currently under investigation in human clinical trials for the treatment of soil-transmitted helminths and possibly *Onchocerca volvulus*. Emodepside targets the calcium-activated voltage-gated potassium slowpoke 1 (SLO-1) channels of presynaptic nerves of pharynx and body wall muscle cells of nematodes leading to paralysis, reduced locomotion and egg laying, starvation, and death. Emodepside also has activity against *Drosophila melanogaster* SLO-1 channels. Orthologous SLO-1 genes are present in *Anopheles gambiae* and *Aedes aegypti*, suggesting that emodepside may have activity against mosquitoes.

**Methods:**

Both *Anopheles dirus* and *Ae. aegypti* were blood-fed emodepside across a range of concentrations (1–10,000 nM) and mosquito survival was monitored for 10 days. Co-feeding experiments were also performed with *An. dirus* blood fed ivermectin at the concentrations that kills 25% (LC_25_) and 50% (LC_50_) of mosquitoes with and without emodepside at clinical peak concentration in humans (C_max_) and five times the C_max_, and mosquito survival was monitored for 10 days.

**Results:**

Emodepside had weak mosquito-lethal effects in *An. dirus* but none observed in *Ae. aegypti* at the concentrations evaluated. The *An. dirus* emodepside LC_50_ was 4,623 [4,159–5,066] ng/ml which is > 100-fold greater than the peak concentrations seen in human. The ivermectin and emodepside co-feed experiment with *An. dirus* did not indicate any altered effect of ivermectin on mosquito survival when emodepside co-fed at human C_max_ or five times that of the human C_max_.

**Conclusions:**

Emodepside was not lethal to *An. dirus* at human-relevant concentrations and had no effect on *Ae. aegypti* survival. Thus, mass distribution of emodepside does not appear to be a potential tool for vector-borne disease control. Emodepside induced mortality in *An. dirus* does suggest that the SLO-1 channel could be a potential target for novel vector control and may warrant further investigation.

## Background

Emodepside, is a semi-synthetic cyclooctadepsipeptide, used in veterinary medicine to treat a broad range of gastrointestinal nematodes, and has a wide range of activity against filarial nematodes [1–3]. A fungus, *Rosellinia* spp. PF1022, naturally produces eight different cyclooctadepsipeptides, and the semisynthetic emodepside (*i.e.* PF1022-221, BAY 44–4400) is produced by the addition of two morpholine rings to the parent 24-membered PF1022A [4]. Emodepside has been evaluated in several healthy human pharmacokinetic trials with a demonstrable safety profile [5, 6]. Similar to ivermectin, emodepside has been shown to have neurological adverse effects in dog breeds with homozygous MDR-1 mutants which are P-glycoprotein deficient [7]. Emodepside is effective against human *Trichuris trichiura* and hookworm infections [8] and clinical trials are currently investigating effectiveness against *Onchocerca volvulus* [9].

Emodepside targets the calcium-activated voltage-gated potassium slowpoke 1 (SLO-1) channels of nematode presynaptic nerves of pharynx and body wall muscle cells. SLO-1 channels are composed of four subunits with seven transmembrane helixes which regulate the rapid repolarization of cells after the depolarizing action potential. Emodepside binds SLO-1 channels, activating the channel to allow influx of potassium causing relaxation of nematode pharynx and/or body wall muscles which can lead to starvation, paralysis, inhibition of egg laying, and death [10–12]. Interestingly, ivermectin- and multi-drug resistant *Haemonchus contortus* in sheep, *Cooperia oncophora* in cattle [13], and *Ancylostoma caninum* in dogs [14] are all susceptible to emodepside, suggesting that this novel class of anthelmintics could be used to treat drug-resistant veterinary and human nematodes.

SLO-1 channels are present in the fruit fly, *Drosophila melanogaster* [12], and orthologs are found in both *Anopheles gambiae* and *Aedes aegypti* [3]. The SLO-1 channel of *D. melanogaster* was transfected into a Chinese Hamster Ovary cell line and subjected to whole cell voltage-clamp electrophysiology demonstrating that *D. melanogaster* SLO-1 channels are activated by emodepside and calcium under increasing voltage exposure [12]. Thus, it is possible that emodepside is lethal to mosquitoes. This opens the potential use of emodepside in human or animal mass drug administration for the control of vector-borne diseases, such as malaria and Dengue. The potential mosquito-lethal effect of emodepside against *An. dirus*, a primary malaria vector in the Greater Mekong Subregion, and *Ae. aegypti*, the primary vector of Dengue, Zika, Chikungunya, and Yellow Fever viruses was investigated.

## Methods

### Mosquitoes

Mosquitoes were reared at the Insecticide Research Unit at the Department of Medical Entomology, Faculty of Tropical Medicine, Mahidol University in Bangkok, Thailand. *Anopheles dirus *sensu stricto (*s.s*.)(Kaw Mai Khaw strain) were produced as described previously [15]. *Aedes aegypti* (Lamplaimas strain) were produced as described previously [16]. Mosquitoes were reared in the insectary at 28 ± 2 °C, 80 ± 10% relative humidity, and 12 h light: 12 h dark photoperiod.

Adult mosquitoes used for experiments were provided 5% sugar solution mixed with 5% multivitamin syrup solution for the first 48 h post emergence and then 10% sucrose solution ad libitum until prepared for experiments. Mosquitoes for experiments were 5–7 days post emergence at the time of blood feeding. The mosquitoes were gently transferred via aspiration to 0.5 L cylindrical cardboard containers sealed with mesh screen on top, and each container held 35–40 mosquitoes. Mosquitoes were maintained in an upright incubator at 25 ± 1 °C and 80 ± 10% humidity with a 12 h light:12 h dark photoperiod. Mosquitoes were sugar-starved with access to water from 16 to 20 h before their blood meal.

### Mosquito blood meal preparation

Whole blood was collected from healthy volunteers on the day of each mosquito membrane feed. Volunteers were screened to ensure they were not taking any medications (*e.g.* CYP3A4 inhibitors) or vitamin supplements (*e.g.* St. John’s wort) that could potentially inhibit emodepside or ivermectin metabolism in the mosquito. Blood was drawn into sodium heparin tubes.

Emodepside and ivermectin were obtained from Sigma-Aldrich (St. Louis, Missouri, USA). Compounds were dissolved in dimethylsulfoxide (DMSO) to a concentration of 2 mg/ml and frozen at − 20 °C until mosquito feeding experiments. Frozen stock solutions of compounds were thawed and serial dilutions were made in human AB + plasma using glass amber vials. For emodepside only experiments, the final plasma solution (10 μl) was mixed with blank whole blood (990 μl) to reach the final concentration desired for mosquito membrane feeding assays (1–10,000 nM; 1.1–11,193.9 ng/ml). For emodepside and ivermectin co-feed experiments, the final plasma solution of emodepside (10 μl) and the final plasma solution of ivermectin (10 μl) was mixed with blank whole blood (980 μl) to reach the final concentration desired for mosquito membrane feeding assays. Ivermectin was blood-fed to *An. dirus* at the lethal concentration that kills 25% (LC_25_ = 4 ng/ml) or 50% (LC_50_ = 6 ng/ml) of mosquitoes [15], and mixed with no emodepside or emodepside at human C_max_ (30 ng/ml), or five times the C_max_ (150 ng/ml) after human adult treatment with 20 mg [5]. Any blood sample prepared for mosquito feeding contained < 1% organic solvent. Control blood meals were prepared, consisting of previously frozen DMSO aliquots without compounds and diluted in plasma to match the highest ratio of DMSO to blood in the compound-containing blood meals.

### Mosquito membrane feeding and mortality assays

At each mosquito membrane feed, whole blood mixed with the compounds were provided to groups of 35–40 *An. dirus* and 25–40 *Ae. aegypti* mosquitoes via membrane feeders warmed to 37 °C. Mosquitoes were allowed to blood feed for up to 30 min. After membrane feeding, up to 30 blood-fed mosquitoes per container were gently transferred via aspiration to clean cardboard containers (0.5 L). After the blood meal, mosquitoes were maintained in an incubator at 25 ± 1 °C and 80 ± 10% humidity with a 12 h light: 12 h dark photoperiod, and provided 10% sucrose ad libitum. Mosquito survival was monitored daily for 10 days and any dead mosquitoes were removed by aspiration and recorded. Ten days after the blood meal any remaining mosquitoes were recorded as alive and then frozen.

### Statistical analyses

The LC_50_ and LC_90_ of mosquitoes from emodepside experiments were estimated using a normalized concentration–response analysis (IC_50_ and Hill), assuming a maximum of 100% mosquito mortality and an estimated baseline mosquito mortality (*i.e.*, mosquito mortality at zero drug concentration). Mosquito survival curves for *An. dirus* fed for each ivermectin concentration (LC_25_, LC_50_) with no emodepside, emodepside at C_max_, or emodepside at five times the C_max_ were compared to using Log-Rank survival curve analysis (Mantel-Cox method). All mosquito survival analyses were performed with GraphPad Prism v.10.2 (GraphPad Software Inc, San Diego, CA, USA).

## Results

### Impact of emodepside on mosquito mortality

**Fig. 1:**
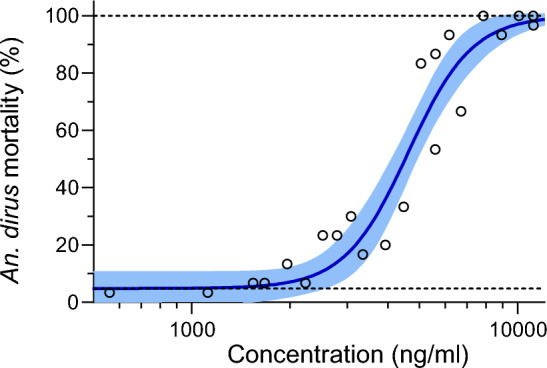
*Anopheles dirus* mortality results when blood-fed emodepside in human blood. Circles represent cumulative mosquito mortality at 10 days after blood meal ingestion. The solid blue line represents the mean concentration–response relationship and the shaded area represents the 95% confidence interval associated with the nonlinear fit. Dashed black lines represent the fixed maximum effects of 100% mortality and the estimated minimum effect associated with baseline mortality observed from control mosquitoes

Three replicates with a total of 900 *An. dirus* mosquitoes, blood-fed emodepside across a range of concentrations (1—10,000 nM; 1.1–11,193.9 ng/ml), were used to calculate the LC_50_ and LC_90_ values for emodepside (Fig. 1). For *An. dirus*, the resulting emodepside LC_50_ = 4,623 [4,159–5,066] ng/ml and LC_90_ = 7,578 [6,317–9,359] ng/ml [95% confidence intervals]. Baseline mortality was low, at an estimated 6.7%.

Two replicates with a total of 598 *Ae. aegypti* were blood-fed a range of emodepside concentrations (1—10,000 nM; 1.1–11,193.9 ng/ml) with no observable mosquito mortality at any concentration, indicating that emodepside was not lethal to *Ae. aegypti* and, therefore, neither an LC_50_ nor LC_90_ value could be generated.

### Impact of ivermectin and emodepside co-feeds on mosquito mortality

**Fig. 2:**
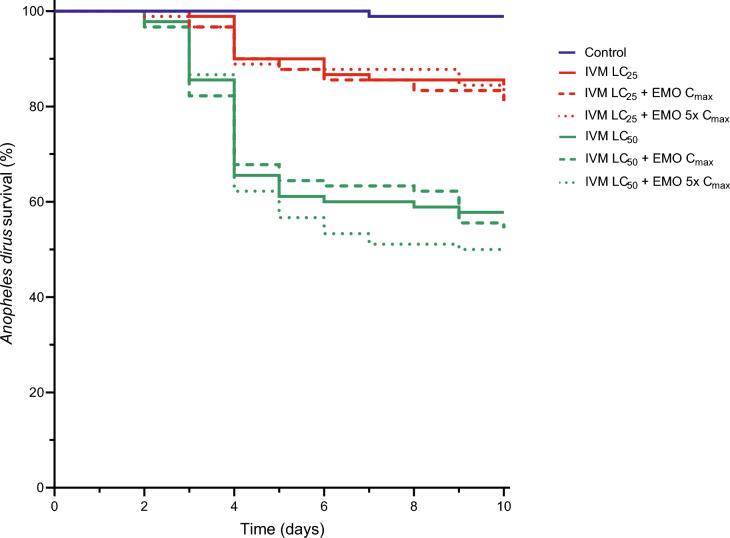
*Anopheles dirus* survival following ingestion of ivermectin and emodepside. Survival results when *An. dirus* was blood-fed no drug control (blue line) or ivermectin (IVM) at the LC_25_ (red lines) or LC_50_ (green lines) and when co-fed with emodepside (EMO) at human C_max_ (dashed lines) or five times the human C_max_ (dotted lines)

Three replicates with a total of 630 *An. dirus* mosquitoes were used to evaluate the interaction of emodepside and ivermectin on *An. dirus* survival when ivermectin at the LC_25_ (4 ng/ml) or LC_50_ (6 ng/ml) was co-fed with or without emodepside at human C_max_ (30 ng/ml) or five times the C_max_ (150 ng/ml) (Fig. 2). As expected, there were significant mortality differences when mosquitoes ingested no drug control (blue line) compared to any combination of ivermectin and/or emodepside (χ^2^ = 98.39, P < 0.0001). However, there were no significant mortality differences when mosquitoes ingested ivermectin LC_25_ compared to ivermectin LC_25_ plus emodepside at C_max_ or five times the C_max_ (χ^2^ = 0.3706, P = 0.8308) (red lines). Similarly, there were no significant mortality differences when mosquitoes ingested ivermectin LC_50_ compared to ivermectin LC_50_ plus emodepside at C_max_ or five times the C_max_ (χ^2^ = 0.8395, P = 0.6572) (green lines).

## Discussion

This is the first investigation of an agent that targets the SLO-1 channel in mosquitoes. Interestingly, there are orthologous SLO-1 genes in both *An. dirus* and *Ae. aegypti*, but emodepside showed only a weak mosquito-lethal effect in *An. dirus* and no mosquito-lethal effect in *An. aegypti*. In *An. dirus*, the estimated emodepside LC_50_ was 4,623 [4,159–5,066] ng/ml (Fig. 1), which is well above the attainable human peak concentration. A clinical trial determined that the ideal adult dose for the treatment of soil-transmitted helminths is 20 mg [8], and healthy adults treated at this dose showed emodepside peak concentrations at approximately 30 ng/ml [5]. While emodepside does not have mosquito-lethal effect in *An. dirus* at human-relevant concentrations, this work does highlight that molecules that target the SLO-1 channel can modulate mosquito survival, providing an alternative target for vector control worthy of further investigation.

In veterinary medicine, emodepside is frequently combined with other drugs, such as praziquantel, toltrazuril, or tigolaner, so it is possible that emodepside could be co-administered with other drugs in humans. Ivermectin is the only endectocide approved for human use and is under investigation as a possible malaria control tool as both humans and animals treated with ivermectin are lethal to *Anopheles* mosquitoes [17]. Since ivermectin and emodepside would target two different channels in the mosquito, glutamate-gated chloride ion channels and SLO-1 channels respectively, this could lead to a synergistic outcome on mosquito survival reduction. To determine if emodepside enhanced the mosquito-lethal effect of ivermectin, *An. dirus* were blood-fed concentrations of ivermectin at the LC_25_ (4 ng/ml) and LC_50_ (6 ng/ml) [15] with or without emodepside at human C_max_ (30 ng/ml) or 5 × C_max_ (150 ng/ml). However, emodepside at these concentrations did not alter ivermectin induced mosquito-lethal outcomes (Fig. 2). These co-feed experiments were not performed with *Ae. aegypti* as ivermectin does not have mosquito-lethal effects in this mosquito species at human-relevant concentrations [18].

## Conclusions

Emodepside was not lethal to *An. dirus* at clinically relevant concentrations and showed no lethal effects in *Ae. aegypti.* Co-administration of emodepside with ivermectin did not alter the mosquito-lethal effects of ivermectin. While emodepside does not appear relevant for malaria or arbovirus control, this work highlights that the SLO-1 channel is a possible target for *Anopheles* vector control which may warrant further investigation.

## Data Availability

Data is available upon reasonable request to the authors.

## Abbreviations

SLO-1: Calcium-activated voltage-gated potassium slowpoke 1
LC_50_: Lethal concentration that kills 50% of mosquitoes
C_max_: Peak concentration in humans
DMSO: Dimethylsulfoxide
